# A Rare Case of Pyopneumothorax Due to *Streptococcus intermedius* in a Previously Healthy Woman

**DOI:** 10.1155/crpu/1665286

**Published:** 2025-10-23

**Authors:** Mahshid Zohouri, Annam Zahid, Anthony Silvestre, Kayhon Rabbani, Karina Dargan, Justin Thomas

**Affiliations:** ^1^Department of Internal Medicine, Eisenhower Medical Center, Rancho Mirage, California, USA; ^2^Department of Internal Medicine, University of California San Francisco, Fresno, California, USA; ^3^Department of Internal Medicine, University of California Riverside School of Medicine, Riverside, California, USA; ^4^Department of Pulmonology and Critical Care, Eisenhower Medical Center, Rancho Mirage, California, USA

## Abstract

Pyopneumothorax secondary to *Streptococcus intermedius* pneumonia contributed to the clinical deterioration and death of a previously healthy adult. This rare case emphasizes the potential for severe complications from common oral flora and the importance of prompt diagnosis and aggressive treatment of pyopneumothorax.

## 1. Background

Hydropneumothorax, a collection of fluid and air in the pleural space, is a rare circumstance that often occurs as a complication of chest trauma, malignancy, infection, and some rheumatologic diseases. When the hydropneumothorax has purulent fluid, it is referred to as an empyema pneumothorax or simply pyopneumothorax. *Mycobacterium tuberculosis*, *Staphylococcus aureus, Pseudomonas aeruginosa, Escherichia coli*, and *Streptococcus* species (sp.) are among the most common causes of pyopneumothorax [1].

*Streptococcus intermedius,* as a part of the *Streptococcus anginosus* group (SAG), also known as *Streptococcus milleri*, is mostly recognized as a part of the oral microbiota. However, it has been reported to cause severe infections, including brain and liver abscesses as well as thoracic empyema [2]. Here, we present a previously healthy female who was admitted to the hospital with pyopneumothorax due to *S. intermedius,* underwent thoracic surgery, but ultimately did not survive her hospitalization.

## 2. Case Presentation

A 52-year-old Caucasian woman with no known past medical history presented to the emergency room (ER) with shortness of breath (SoB) and a productive cough for 1 month. Her review of systems was significant for weight loss of over 10 kg in 3 months, and swelling of the legs, but negative for fevers, chills, or any other signs of infection. She had received outpatient treatment with azithromycin and a short course of corticosteroids (oral prednisone 20 mg × 5 days); however, her symptoms did not improve. The patient had worked in a plant nursery where she was exposed to pollen. She had no history of smoking or drug use.

Upon presentation, she was febrile at 102.5 Fahrenheit (39.2°C), saturating 95% on 15 L of oxygen/min via a nonrebreather mask, heart rate 124, and blood pressure 150/75. The lung auscultation was notable for wheezing and rales in the right middle and lower lung fields. On physical examination, she was also found to have bilateral lower extremity 3+ pitting edema. Initial labs showed white blood count of 9.9 K/uL (reference range (RR): 4.5–11.0 K/uL), 16% band cells (RR: 0%–3%), creatinine 0.6 mg/dL (RR: 0.5–1.1 mg/dL), C-reactive protein 22 mg/dL (RR: < 1.0 mg/dL), erythrocyte sedimentation rate 83 mm/h (RR: 0–25 mm/h), and normal liver function tests.

**Figure 1:**
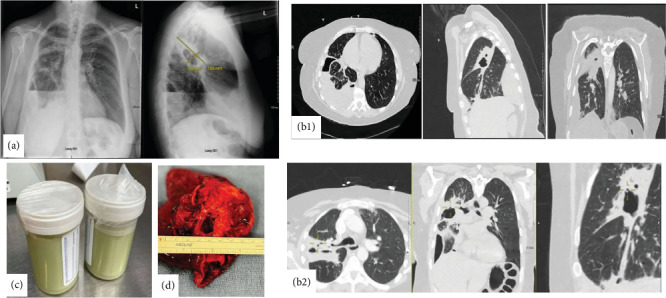
(a) Initial chest X-ray upon presentation showing extensive heterogeneous right lung opacity, which appears to be a thick-walled cavitary lesion about 4.8 × 10.5 cm. Moderate right loculated hydropneumothorax at the lung base with associated air-fluid level, concerning for empyema. (b) b1: A thick-walled cavitary consolidation or lesion in the posterior right upper lobe. There is a complex moderate right pleural effusion with air-fluid levels, concerning for empyema. There is extensive parabronchial thickening in the right lung. b2: CT chest after chest tube insertion; the yellow marker is pointing to a bronchopulmonary fistula, which was the reason for her continuous air leakage. (c) Frank pus after inserting the chest tube. (d) Right upper lobe abscess after lobectomy.

Chest X-ray (CXR) showed hydropneumothorax, and CT chest with contrast revealed a small burden of left-sided acute pulmonary embolism (PE), a large thick-walled irregular cavitary consolidation or mass in the right upper lobe (RUL) with an internal air-fluid level, and a moderate right-sided loculated hydropneumothorax/empyema (Figure 1a,b). An ultrasound of the bilateral lower extremities revealed bilateral nonocclusive, acute appearing deep venous thrombi (DVT) in the right popliteal, left superficial femoral, and left popliteal veins.

The patient had a history of severe penicillin allergy, so she was started on broad-spectrum antibiotic therapy with intravenous vancomycin, levofloxacin, and metronidazole for treatment of sepsis secondary to a parapneumonic effusion. Heparin infusion was also started for the PE. On the second day of admission, she underwent percutaneous chest tube placement, and malodorous frank pus (Figure 1c) was aspirated. Cytologic analysis showed no malignant cells and marked acute inflammation, consistent with an empyema. She received intrapleural fibrinolytics (alteplase and dornase alpha-tPA) for a total of six doses. On Day 2, she underwent bronchoscopy with bronchoalveolar lavage (BAL) and biopsy.

Due to persistent fevers and worsening leukocytosis, antibiotics were changed to vancomycin and meropenem. Pleural fluid cytology was negative on two occasions, and culture results showed moderate growth of *Streptococcus intermedius* (minimum inhibitory concentration (MIC) susceptible to ceftriaxone and penicillin). Although the isolate also showed susceptibility to vancomycin and levofloxacin via the Kirby–Bauer disk diffusion, MIC values were not provided. BAL grew a few colonies of *Candida albicans*, which were not felt to be clinically significant. Given her clinical instability and penicillin allergy, vancomycin was discontinued, and meropenem was continued for its broad coverage.

A maxillofacial CT did not show findings to indicate sinusitis or abscesses. CXR and CT scans were repeated, which showed interval improvement in empyema but were also remarkable for extensive subcutaneous emphysema, which, in conjunction with a persistent air leak in the chest tube, was attributed to be secondary to a bronchopleural fistula (Figure 1b, b2). Cardiothoracic surgery was consulted, and on Day 6, the patient underwent a right video-assisted thoracoscopy and thoracotomy, which revealed a large RUL abscess necessitating a right upper lobectomy followed by a tube thoracostomy (Figure 1d). Abscess cultures later grew *Corynebacterium tuberculostearicum*, but results were finalized after the patient's passing. Given the delayed growth and its usual role as a skin commensal, it was not considered the primary pathogen.

After surgery, she was observed in the intensive care unit and was successfully extubated within 24 h. The patient was doing well for a few hours but developed an unexplained cardiac arrest, and all resuscitation efforts were unsuccessful. She passed away after 7 days of hospitalization. Her family did not wish to proceed with an autopsy; therefore, her cause of death remains unclear.

## 3. Discussion

Pyopneumothorax, defined as an accumulation of pus and air in the pleural cavity, is associated with high morbidity and mortality. Although *M. tuberculosis* remains a leading cause globally, bacterial pathogens, including *Streptococcus* species, have been increasingly recognized. Data about the prevalence of hydropneumothorax, especially pyopneumothorax, are very limited, but it has been shown to occur as a complication of necrotizing pneumonia leading to a bronchopleural fistula [1].

SAG includes *S. anginosus, S. intermedius*, and *S. constellatus* species [3]. Although *S. intermedius* is usually a benign oral flora species, it can act as an opportunistic pathogen, causing severe infections such as brain abscesses, liver abscesses, and empyema [2]. *S. intermedius*' involvement in pleuropulmonary infection (PPI), including parapneumonic effusion or empyema, is very rare [4]. It has been found as a causal organism in 14% of respiratory tract infection cases caused by the *S. milleri* group [5]. However, among the different pulmonary infections, it is more prevalent as a causative pathogen in abscess and empyema compared to pneumonia, 13%–44% versus 2%–5%, respectively. Moreover, *S. milleri* group species are the most common organisms in primary empyema, along with *Fusobacterium nucleatum* [6, 7].

In this case, *S. intermedius* was isolated early from pleural fluid cultures, supporting its role as the primary pathogen. While exact colony-forming unit (CFU) numbers were not available in the final report, the culture yielded a moderate growth of *S. intermedius* in a sterile compartment. This, combined with the absence of other pathogenic organisms at the time of collection, supports its clinical relevance. Since the pleural fluid was obtained after the initiation of antibiotics, we cannot entirely exclude the possibility of a polymicrobial infection. Although *C. tuberculostearicum* was later identified from the pulmonary abscess, its clinical relevance is uncertain; it is a common skin colonizer and potential contaminant in pulmonary specimens. Furthermore, clinical presentation, imaging findings, and culture results were consistent with invasive PPI. Susceptibility testing confirmed sensitivity to beta-lactam antibiotics; however, due to the patient's clinical deterioration, broad-spectrum coverage was continued rather than narrowed, reflecting a stewardship limitation in critically ill patients. Her clinical course was further complicated by extensive DVT and PEs, likely related to immobility over the preceding weeks due to severe respiratory distress on exertion. Anticoagulation was held intermittently for procedural reasons, including chest tube placement and in the perioperative period. During the resuscitation, a recurrent PE was suspected; however, push-dose tPA was not administered due to recent thoracic surgery and bloody output from the chest tube, which posed a significant bleeding risk. We suspect that the patient may have suffered a fatal PE, which could have contributed to her rapid deterioration and death, although this could not be confirmed as the patient's family declined an autopsy.

There are few reports of pyopneumothorax caused by *S. milleri* group, and this case would be the fourth report of SAG and the first report of *S*. *intermedius* [1, 8, 9]. One of the reasons that we are seeing an increasing number of reports about Group A *Streptococcus* (GAS) pathogenicity might be that it has been called *Viridans streptococci* for years; therefore, the clinical manifestation might be underrepresented in the literature [3].

This case is unique because pyopneumothorax secondary to *S. intermedius* is exceedingly rare, and prior reports more often involve individuals with risk factors such as dental disease, diabetes, or alcohol use [2, 4]. Our patient, notably, had no identifiable risk factors commonly associated with being immunocompromised, and her history did not raise suspicion for underlying immunodeficiency. Although the patient declined human immunodeficiency virus (HIV) testing, given the slow healing and poor response to usual treatment, she clinically met indications for further immunodeficiency evaluation; however, this was not performed during hospitalization, which represents a limitation of this report. This case highlights that *S. intermedius* can cause life-threatening infections even in otherwise healthy individuals.

The rapid progression of infection despite early intervention underscores the virulent potential of *S. intermedius* and the importance of maintaining a high index of suspicion for aggressive organisms in complicated PPI. Further research is needed to better understand the pathogenic mechanisms and clinical predictors associated with severe PPIs caused by *S. intermedius*.

## 4. Conclusion

This case highlights the pathogenic potential of *S. intermedius*, a typically benign oral commensal, to cause severe PPI even in previously healthy individuals. Recognition of *S. intermedius* as an aggressive pathogen in pyopneumothorax is critical for early diagnosis and management. Greater awareness and further research into the virulence factors of *S. intermedius* may improve outcomes in patients with complicated pleural space infections.

## Data Availability

The data that support the findings of this study are available on request from the corresponding author.

## References

[1] Che Rahim M. J., Mohammad N., Wan Ghazali W. S. (2016). Pyopneumothorax Secondary to Streptococcus milleri infection. *BML Case Reports*.

[2] Issa E., Salloum T., Tokajian S. (2020). From Normal Flora to Brain Abscesses: A Review of Streptococcus intermedius. *Frontiers in Microbiology*.

[3] Pilarczyk-Zurek M., Sitkiewicz I., Koziel J. (2022). The Clinical View on Streptococcus anginosus Group - Opportunistic Pathogens Coming Out of Hiding. *Frontiers in Microbiology*.

[4] Tasleem A., Mahmood A., Sharma R. (2021). Streptococcus intermedius Pleuropulmonary Disease: A Not So Commonly Seen Radiological Picture. *Cureus*.

[5] Wong C. A., Donald F., Macfarlane J. T. (1995). Streptococcus milleri Pulmonary Disease: A Review and Clinical Description of 25 Patients. *Thorax*.

[6] Noguchi S., Yatera K., Kawanami T. (2014). Pneumonia and Empyema Caused by Streptococcus intermedius that Shows the Diagnostic Importance of Evaluating the Microbiota in the Lower Respiratory Tract. *Internal Medicine*.

[7] Iskandar S. B., al Hasan M. A., Roy T. M., Jr Byrd R. P. (2006). Streptococcus intermedius: An Unusual Cause of a Primary Empyema. *Tennessee Medicine: Journal of the Tennessee Medical Association*.

[8] Eller P., Theurl I., Koppelstaetter F., Weiss G. (2006). Pyopneumothorax Due to Streptococcus milleri. *Wiener Klinische Wochenschrift*.

[9] Zhang Z., Xiao B., Liang Z. (2020). Successful Treatment of Pyopneumothorax Secondary to Streptococcus constellatus Infection With Linezolid: A Case Report and Review of the Literature. *Journal of Medical Case Reports*.

